# Supplementary material for: Learning interpretable dynamics of stochastic complex systems from experimental data
Source: Nat Commun. 2024 Jul 17;15:6029. doi: 10.1038/s41467-024-50378-x (PMC11254936; doi:10.1038/s41467-024-50378-x)
Supplement: Supplementary file 1 — Supplementary Information [file 41467_2024_50378_MOESM1_ESM.pdf]

Supplementary Information for

Learning interpretable dynamics of stochastic complex systems from experimental data

Contents

I Langevin graph network approach (LaGNA) 2

A Learning with message passing scheme . . . 2

B Penetrating neural network modules . . . 4

II Learning stochastic networked dynamics from synthetic data 6

A Synthetic complex networks . . . 6

B Inference results of stochastic network dynamics . . . 6

B.1 Stochastic Lorenz dynamics on complex networks . . . 7

B.2 Stochastic Rössler dynamics on complex networks . . . 7

B.3 Stochastic Hindmarsh-Rose neuronal dynamics on complex networks . . . 9

B.4 Second-order stochastic dynamics of Vicsek flocking model . . . 10

III Learning from experimental data 11

A Interpretable dynamics of real bird flocks . . . 11

B Interpretable dynamics of tau pathology propagation . . . 11

C Details of the penetration procedure . . . 13

IV Robustness test of LaGNA 19

V Additional comparisons 20

A Description of baseline methods . . . 20

B Learning from noisy data with a denoising preprocess . . . 21

C Inferring single-node stochastic dynamics from noisy data . . . 23

D Method comparisons for AD propagation data . . . 23

Supplementary References 25

I Langevin graph network approach (LaGNA)

A Learning with message passing scheme

The core structure of the Langevin graph network approach (LaGNA) for identifying the three components of stochastic differential equations (SDEs) is shown below, with the parameters also displayed in Sup. Table 1-3. The LaGNA uses a dividing-and-conquering strategy, providing explicit guidance for the regression process with comprehensible sub-problems. The construction of the message-passing scheme plays a crucial role in propagating information and capturing the interaction dynamics. Specifically, the non-zero element A_{ij} of the adjacency matrix A represents an edge between nodes j and i. It signifies the flow of information from node j to node i. To describe the characteristics of the edges, an edge feature function $\hat{g}(\mathbf{x}_i, \mathbf{x}_j, A_{ij})$ is introduced (with one dimension as example). This function takes as input the feature vectors of the connected nodes ($\mathbf{x}_i$ and $\mathbf{x}_j$) and the edge weight A_{ij}. It produces a representation of the interaction or relationship between the nodes. To capture the combined effect of all source nodes j on a target node i, an aggregation function denoted by $\square_{j=1}^{n}$ is employed. This aggregation function combines the edge features $\hat{g}(\mathbf{x}_i, \mathbf{x}_j, A_{ij})$ for all neighboring nodes j into a single representation. Differentiable aggregation functions, such as "sum," "mean", or "max," can be used. In the case of LaGNA, the "sum" aggregation function is adopted. Consequently, the change of $\mathbf{x}_i$ can be described as $\mathrm{d}\mathbf{x}_i = \hat{f}(\mathbf{x}_i) + \square_{j=1}^{n}\hat{g}(\mathbf{x}_i, \mathbf{x}_j, A_{ij}) + \hat{\phi}(\mathbf{x}_i)\mathrm{d}\mathbf{W}_t$, where $\square = \sum$. Functions $\hat{g}(\cdot)$, $\hat{f}(\cdot)$ and $\hat{\phi}(\cdot)$ are implemented using multi-layer perceptrons (MLPs) capturing complex relationships and nonlinear dynamics in a flexible and learnable manner.

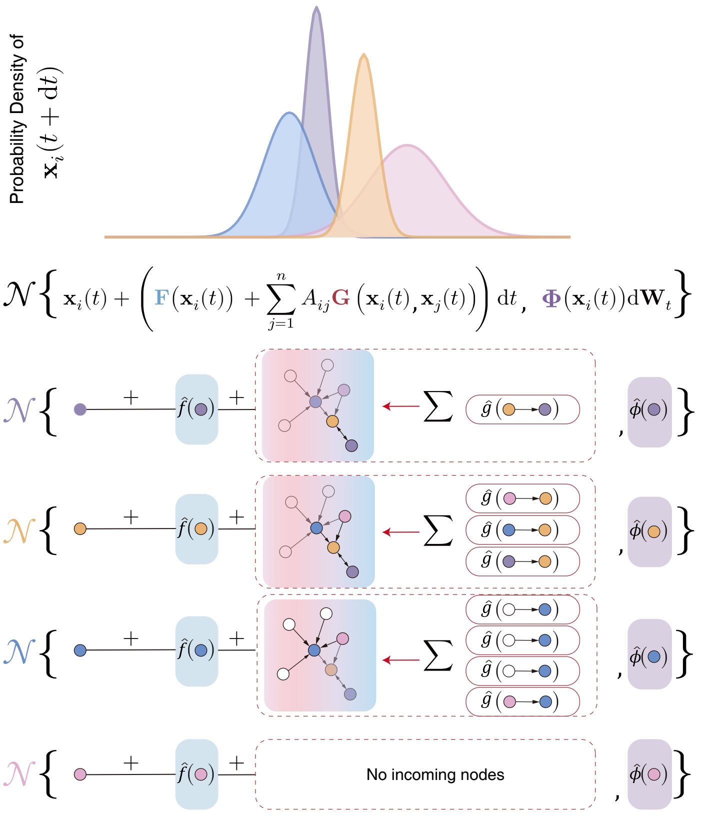

Supplementary Figure 1: Illustration of LaGN architecture. For each node, the probability density distribution is represented by the mean estimated by aggregation of $\hat{g}(\cdot)$ and $\hat{f}(\cdot)$, and variance estimated by diffusion intensity estimator $\hat{\phi}(\cdot)$.

Supplementary Table 1: Parameters of $\hat{g}(\cdot)$

$\hat{g}(\cdot)$	Input	Layers	Hidden neurons	Output	Activation functions
Lorenz (unweighted)	2	3	100	1	ReLU
Rossier (weighted)	2	3	100	1	ReLU
HR (heterogeneous)	2	3	100	1	ReLU
Vicsek (2nd-order, g_a)	1	3	100	1	ReLU
Vicsek (2nd-order, g_c)	1	3	100	1	ReLU
Empirical flocks (g_a)	1	3	100	1	ReLU
Empirical flocks (g_c)	1	3	100	1	ReLU
Pathological tau (g_{retro})	2	4	200	1	ReLU
Pathological tau (g_{antero})	2	4	200	1	ReLU
Pathological tau (g_{Euc})	2	4	200	1	ReLU

Supplementary Table 2: Parameters of $\hat{f}(\cdot)$

$\hat{f}(\cdot)$	Input	Layers	Hidden neurons	Output	Activation functions
Lorenz (unweighted)	3	3	100	1	ReLU
Rossier (weighted)	3	3	100	1	ReLU
HR (heterogeneous)	3	3	100	1	ReLU
Vicsek (2nd-order)	1	3	100	1	ReLU
Empirical flocks	2	3	100	1	ReLU, Sigmoid
Pathological tau (self)	1	3	200	1	ReLU
Pathological tau (time)	1	3	200	1	ReLU

Supplementary Table 3: Parameters of $\hat{\phi}(\cdot)$

$\hat{\phi}(\cdot)$	Input	Layers	Hidden neurons	Output	Activation functions
Lorenz (unweighted)	3	2	100	1	ReLU, softplus
Rossier (weighted)	3	2	100	1	ReLU, softplus
HR (heterogeneous)	3	2	100	1	ReLU, softplus
Vicsek (2nd-order)	6	2	100	1	ReLU, softplus
Empirical flocks	6	1	100	1	ReLU, softplus
Pathological tau	1	2	100	1	ReLU, softplus

B Penetrating neural network modules

We constructed three comprehensive libraries, L_F, L_G, and L_Φ, for self, interaction, and diffusion dynamics functions, respectively. These libraries include polynomial, trigonometric, exponential, fractional, rescaling, and various activation functions as listed in Supplementary Table 4. To obtain the diffusion functions, we simply take the absolute value of L_F. The tables mentioned above were adapted from[S1].

*sigmoid: indicates a variety of sigmoid functions $\frac{1}{1+e^{-\alpha(x-\beta)}}$, where $\alpha \in [1:1:10]$ and $\beta \in [0:1:10]$.

†$\gamma \in [0:1:10]$.

Supplementary Table 4: Elementary functions Library for self and interaction dynamics

Functions	Polynomial	Trigonometric	Exponential	Fractional	Activation	Rescaling
$\boldsymbol{\Theta}_F = L_F(\mathbf{x}_i)$	$\mathbf{x}_i,\ \mathbf{x}_i^2,\ \mathbf{x}_i^3,\ \cdots$	$\sin(\mathbf{x}_i),\ \cos(\mathbf{x}_i),\ \tan(\mathbf{x}_i)$	$e^{\mathbf{x}_i}$	$\frac{1}{\mathbf{x}_i}$	$\{\mathrm{sigmoid}(\mathbf{x}_i)\}^*$, $\tanh(\mathbf{x}_i)$, $\{\frac{\mathbf{x}_i^\gamma}{\mathbf{x}_i^\gamma+1}\}^\dagger$	$\frac{\mathbf{x}_i}{k_i^{\mathrm{in}}}$
$\boldsymbol{\Theta}_G = L_G(\mathbf{x}_j)$	$\mathbf{x}_j,\ \mathbf{x}_j^2,\ \cdots$	$\sin(\mathbf{x}_j)$, $\cos(\mathbf{x}_j),\ \cdots$	$e^{\mathbf{x}_j}$	$\frac{1}{\mathbf{x}_j}$	$\{\mathrm{sigmoid}(\mathbf{x}_j)\}^*$, $\tanh(\mathbf{x}_j)$, $\{\frac{\mathbf{x}_j^\gamma}{\mathbf{x}_j^\gamma+1}\}^\dagger$	$\frac{\mathbf{x}_j}{k_i^{\mathrm{in}}}$
$\boldsymbol{\Theta}_G = L_G(\mathbf{x}_i\mathbf{x}_j)$	$\mathbf{x}_i\mathbf{x}_j,\ (\mathbf{x}_i\mathbf{x}_j)^2,\ \cdots$	$\sin(\mathbf{x}_i\mathbf{x}_j)$, $\cos(\mathbf{x}_i\mathbf{x}_j),\ \cdots$	$e^{\mathbf{x}_i\mathbf{x}_j}$	$\frac{1}{\mathbf{x}_i\mathbf{x}_j}$	$\{\mathrm{sigmoid}(\mathbf{x}_i\mathbf{x}_j)\}^*$, $\tanh(\mathbf{x}_i\mathbf{x}_j)$, $\{\frac{(\mathbf{x}_i\mathbf{x}_j)^\gamma}{(\mathbf{x}_i\mathbf{x}_j)^\gamma+1}\}^\dagger$	$\frac{\mathbf{x}_i\mathbf{x}_j}{k_i^{\mathrm{in}}}$
$\boldsymbol{\Theta}_G = L_G(\mathbf{x}_j-\mathbf{x}_i)$	$\mathbf{x}_j-\mathbf{x}_i,\ (\mathbf{x}_j-\mathbf{x}_i)^2,\ \cdots$	$\sin(\mathbf{x}_j-\mathbf{x}_i)$, $\cos(\mathbf{x}_j-\mathbf{x}_i),\ \cdots$	$e^{\mathbf{x}_j-\mathbf{x}_i}$	$\frac{1}{\mathbf{x}_j-\mathbf{x}_i}$	$\{\mathrm{sigmoid}(\mathbf{x}_j-\mathbf{x}_i)\}^*$, $\tanh(\mathbf{x}_j-\mathbf{x}_i)$, $\{\frac{(\mathbf{x}_j-\mathbf{x}_i)^\gamma}{(\mathbf{x}_j-\mathbf{x}_i)^\gamma+1}\}^\dagger$	$\frac{\mathbf{x}_j-\mathbf{x}_i}{k_i^{\mathrm{in}}}$
$\boldsymbol{\Theta}_G = \mathbf{x}_iL_G(\mathbf{x}_j)$		$\mathbf{x}_i\sin(\mathbf{x}_j)$, $\mathbf{x}_i\cos(\mathbf{x}_j),\ \cdots$	$\mathbf{x}_ie^{\mathbf{x}_j}$	$\frac{\mathbf{x}_i}{\mathbf{x}_j}$	$\{\mathbf{x}_i\mathrm{sigmoid}(\mathbf{x}_j)\}^*$, $\mathbf{x}_i\tanh(\mathbf{x}_j)$, $\{\frac{\mathbf{x}_i\mathbf{x}_j^\gamma}{\mathbf{x}_j^\gamma+1}\}^\dagger$	$\frac{\mathbf{x}_i\mathbf{x}_j}{k_i^{\mathrm{in}}}$
$\boldsymbol{\Theta}_{GA} = L_{GA}(r_{ij})$, $\boldsymbol{\Theta}_{GC} = L_{GC}(r_{ij})$	$r_{ij},\ r_{ij}^2,\ r_{ij}^3,\ \cdots,$	$sin(r_{ij})$, $\cos(r_{ij}),\ \cdots$	$e^{r_{ij}}$	$\frac{1}{r_{ij}}$	$\frac{1-(r_{ij}/2)^3}{1+(r_{ij}/2)^6}$, $\frac{(r_{ij}/2-1)^3}{(1+r_{ij}/2)^6}$, $e^{\frac{-r_{ij}}{3}}$	

II Learning stochastic networked dynamics from synthetic data

To test our framework, we generated stochastic network dynamics based on a variety of stochastic differential equations and three types of network topologies. We used the resulting nodes' time series $\mathbf{x}_i$, where $i \in n$, as input to calculate the numerical first and second derivatives

$$v_i \equiv \dot{x}_i(t) = \frac{x_i(t+\mathrm{d}t) - x_i(t)}{\mathrm{d}t}, \tag{S1}$$

$$a_i \equiv \ddot{x}_i(t) = \frac{x_i(t+\mathrm{d}t) - 2x_i(t) + x_i(t-\mathrm{d}t)}{\mathrm{d}t^2}, \tag{S2}$$

which correspond to velocities and accelerations, respectively, and $\mathrm{d}t = 0.01$ unless otherwise stated.

A Synthetic complex networks

The original directed scale-free network used to simulate time series was generated by the static model with $n = 20$ and $E = 80$, as shown in Sup. Fig. 2a[S2]. To generate the weighted network, we generated 80 random numbers ranging from $(0, 1]$ and randomly assigned them to the existing edges in the unweighted network, as shown in Sup. Fig. 2b. For the signed network, we designed two assignment strategies. The first strategy involved randomly selecting a certain proportion of inhibitory nodes, and the edges that come from these nodes are classified as inhibitory. The second strategy involved randomly selecting a certain proportion of inhibitory edges directly, as shown in Sup. Fig.2c, d.

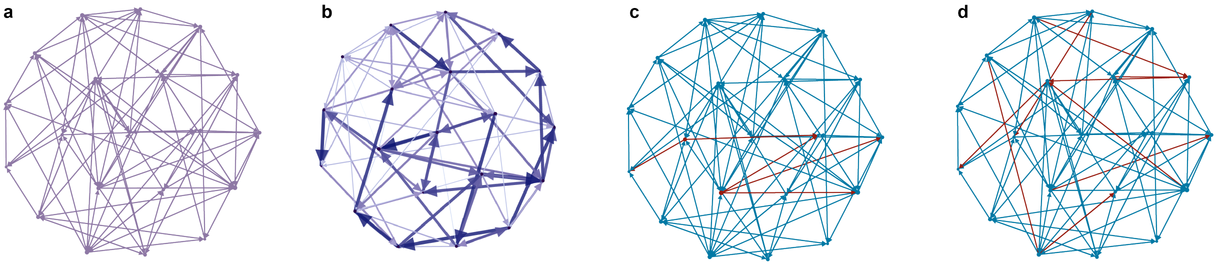

Supplementary Figure 2: **a**.Unweighted network with 20 nodes and 80 directed links, which is the original network for deformation. **b**. Weighted network. **c-d**. signed networks with inhibitory nodes and links, respectively, and indicated by orange nodes and edges.

B Inference results of stochastic network dynamics

To numerically simulate the stochastic dynamics, we adopt the simple Euler-Maruyama scheme to integrate over a very short time $\mathrm{d}t > 0$[S3]:

$$\mathbf{x}_i(t+\mathrm{d}t) = \mathbf{x}_i(t) + (\mathbf{F}(\mathbf{x}_i(t)) + \sum_{j=1}^{n} A_{ij}\mathbf{G}(\mathbf{x}_i(t), \mathbf{x}_j(t)))\mathrm{d}t + \mathbf{\Phi}(\mathbf{x}_i(t))\mathrm{d}\mathbf{W}_t, \tag{S3}$$

where $\mathrm{d}\mathbf{W}_t$ is a vector whose length equals the dimension of the dynamics, and it is normally distributed around zero with variance $\mathrm{d}t$. In this section, we begin by presenting the complete inference of the stochastic Lorenz dynamics as an example and then proceed to show other inference results as well.

B.1 Stochastic Lorenz dynamics on complex networks

First, we simulate stochastic network dynamics governed by canonical Lorenz chaotic dynamics on the unweighted network[S4]

$$\begin{cases} \mathrm{d}x_{i,1} = (ax_{i,2} - (a + \frac{2}{\gamma})x_{i,1} + \epsilon\sum_{j=1}^{n} A_{i,j}x_{j,1})\mathrm{d}t + |\frac{x_{i,1}}{\sqrt{\gamma}}|\mathrm{d}W_{i,1}, \\ \mathrm{d}x_{i,2} = (\rho - x_{i,3})x_{i,1} - (1 + \frac{2}{\gamma})x_{i,2})\mathrm{d}t + |\frac{\rho - x_{i,3}}{\sqrt{\gamma}}|\mathrm{d}W_{i,2}, \\ \mathrm{d}x_{i,3} = (x_{i,1}x_{i,2} - (\beta + \frac{4}{\gamma})x_{i,3})\mathrm{d}t + |\frac{x_{i,2}}{\sqrt{\gamma}}|\mathrm{d}W_{i,3}. \end{cases} \tag{S4}$$

Here, we set $a = 10$, $\gamma = 1$, $\epsilon = 1$, $\rho = 28$, $\beta = 8/3$, and $\mathrm{d}\mathbf{W}_i$ as a d-dimensional random vector distributed around zero with variance $\mathrm{d}t = 0.01$. We generate stochastic trajectories for $T = 100$ with $\mathrm{d}t = 0.01$, meaning that all timestamps are 10000. The 3-dimensional trajectory of the first node in the network is shown in Sup. Fig. 3a.

Using our inference framework, we accurately estimate the self, interaction, and diffusion parts with LaGNA, as shown in Sup. Fig. 3(b-d). We then utilize the two-phase inference to identify the corresponding functions for each part (Sup. Fig. 3(e-g)). The inferred stochastic equation is

$$\begin{cases} \mathrm{d}x_{i,1} = (10.07x_{i,2} - 12.12x_{i,1} + \sum_{j=1}^{n} A_{i,j}x_{j,1})\mathrm{d}t + |1.01x_{i,1}|\mathrm{d}W_{1,t}, \\ \mathrm{d}x_{i,2} = ((27.61 - 0.99x_{i,3})x_{i,1} - 2.95x_{i,2})\mathrm{d}t + |30.5 - 1.09x_{i,3}|\mathrm{d}W_{2,t} \\ \mathrm{d}x_{i,3} = (x_{i,1}x_{i,2} - 6.68x_{i,3})\mathrm{d}t + |1.01x_{i,2}|\mathrm{d}W_{3,t}, \end{cases} \tag{S5}$$

which is accurate and the inaccuracy sMAPE is also shown in Sup. Fig. 3h.

B.2 Stochastic Rössler dynamics on complex networks

To demonstrate our framework's effectiveness in inferring stochastic dynamics on the weighted network, we simulate Rössler oscillators dynamics on the weighted network[S5]:

$$\begin{cases} \mathrm{d}x_{i,1} = (-x_{i,2} - x_{i,3} + \epsilon\sum_{j=1}^{n} A_{i,j}(x_{j,1} - x_{i,1}))\mathrm{d}t + \eta|x_{i,1}|\mathrm{d}W_{1,t}, \\ \mathrm{d}x_{i,2} = (x_{i,1} + 0.35x_{i,2})\mathrm{d}t + \eta|x_{i,2}|\mathrm{d}W_{2,t} \\ \mathrm{d}x_{i,3} = (0.2 + x_{i,3}(x_{i,1} - 5.7))\mathrm{d}t + \eta|x_{i,3}|\mathrm{d}W_{3,t}, \end{cases} \tag{S6}$$

where $\epsilon = 0.5$, and diffusion intensity $\eta = 0.1$. We generate stochastic trajectories in $T = 400$ with $\mathrm{dt} = 0.01$, *i.e.* all timestamps are 40000. The inference stochastic equation is:

$$\begin{cases} \mathrm{d}x_{i,1} = (-1.003x_{i,2} - x_{i,3} + 0.508\sum_{j=1}^{n} A_{i,j}(x_{j,1} - x_{i,1}))\mathrm{d}t + 0.501|x_{i,1}|\mathrm{d}W_{1,t}, \\ \mathrm{d}x_{i,2} = (x_{i,1} + 0.343x_{i,2})\mathrm{d}t + 0.5|x_{i,2}|\mathrm{d}W_{2,t} \\ \mathrm{d}x_{i,3} = (0.211 + 0.992x_{i,3}(x_{i,1} - 5.656))\mathrm{d}t + 0.498|x_{i,3}|\mathrm{d}W_{3,t}. \end{cases} \tag{S7}$$

The inference results, including the estimations of NNs and the inaccuracy, are shown in Sup. Fig. 4:

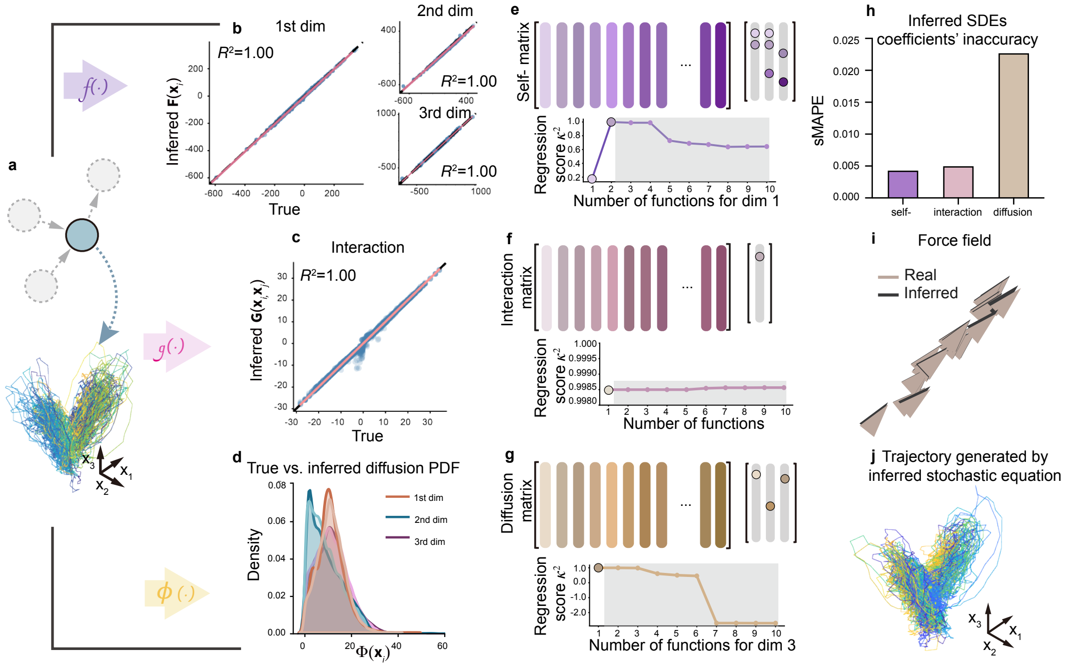

Supplementary Figure 3: Inference of stochastic Lorenz dynamics. **a**. The trajectory of the first node in the network generated by the true dynamics. **b**. Inferred 3-dimensional self parts from NN $\hat{\boldsymbol{f}}(\cdot)$ vs. true values. The R^2 is the square of the Pearson correlation coefficient. **c**. Inferred interaction part from NN $\hat{g}(\cdot)$ vs. true values. **d**. Inferred distribution of 3 dimensions' diffusion intensity vs. the true intensity distribution. **e-g**. Identification of the elementary functions of different dimensions according to the inferred self, interaction and diffusion dynamics with two-phase inference. **h**. Inference inaccuracy sMAPE of the inferred stochastic Lorenz dynamics. **i**. Comparison between the generated force field by inferred equation and the true force field at time $t = 10$. **j**. The 3-dimensional trajectory generated by the inferred stochastic Lorenz equation.

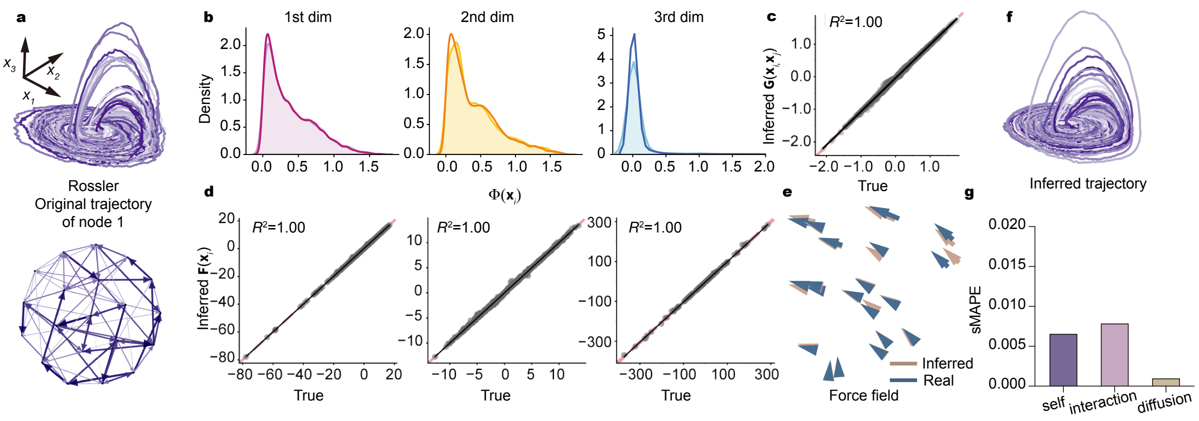

Supplementary Figure 4: **a**. The trajectory simulated by the true stochastic Rössler dynamics and the weighted network topology. **b**. The inferred distribution density of the diffusion intensity vs. true intensity distribution. **c**. The inferred interaction by $\hat{g}(\cdot)$ vs. the true interaction values. **d**. The inferred 3-dimensional self by $\hat{\boldsymbol{f}}(\cdot)$ vs. the true self values. **e**. Comparison between the generated force field by inferred equation and the true force field at time $t = 10$. **f**. The 3-dimensional trajectory generated by the inferred stochastic Rössler equation. **g**. Inference inaccuracy sMAPE of the inferred stochastic Rössler dynamics.

B.3 Stochastic Hindmarsh-Rose neuronal dynamics on complex networks

To test the ability to infer neuronal dynamics, we simulate inhibitory-excitatory edge-composed network dynamics using Hindmarsh-Rose (HR) dynamics[S6]. The signed networks are constructed based on the two strategies described above (Fig. 2c,d). The true stochastic HR dynamics are

$$\begin{cases} \mathrm{d}x_{i,1} = (x_{i,2} - x_{i,1}^3 + 3x_{i,1}^2 - x_{i,3} + I_{ext} + gc\sum_{j=1}^{n} A_{i,j}G(x_{i,1}, x_{j,1}))\mathrm{d}t + \eta|x_{i,1}|\mathrm{d}W_{1,t}, \\ \mathrm{d}x_{i,2} = (1 - 5x_{i,1}^2 - x_{i,2})\mathrm{d}t + \eta|x_{i,2}|\mathrm{d}W_{2,t} \\ \mathrm{d}x_{i,3} = (0.005(4(x_{i,1} + 1.6) - x_{i,3}))\mathrm{d}t + \eta|x_{i,3}|\mathrm{d}W_{3,t}, \end{cases} \tag{S8}$$

where coupling strength $I_{ext} = 3.24$, $gc = 0.15$, and $\eta = 0.1$.

$$G(x_{i,1}, x_{j,1}) = \frac{V_{syn} - x_{i,1}}{1 + \exp(-10(x_{j,1} - 1))} \tag{S9}$$

is interaction function where $V_{syn} = 2$ for excitatory edges and $V_{syn} = -1.5$ for inhibitory edges. We simulate $T = 500$, $\mathrm{d}t = 0.01$ trajectories on the signed network. The simulation results as input, the inferred stochastic equations are:

$$\begin{cases} \mathrm{d}x_{i,1} = (0.998x_{i,2} - 0.993x_{i,1}^3 + 2.994x_{i,1}^2 - 0.982x_{i,3} + 3.204 + gc_i\sum_{j=1}^{n} A_{i,j}G_i(x_{i,1}, x_{j,1}))\mathrm{d}t \\ \qquad + 0.101|x_{i,1}|\mathrm{d}W_{1,t}, \\ \mathrm{d}x_{i,2} = (0.995 - 4.981x_{i,1}^2 - 0.995x_{i,2})\mathrm{d}t + 0.098|x_{i,2}|\mathrm{d}W_{2,t} \\ \mathrm{d}x_{i,3} = (0.031 + 0.019x_{i,1} - 0.005x_{i,3})\mathrm{d}t + 0.098|x_{i,3}|\mathrm{d}W_{3,t}, \end{cases} \tag{S10}$$

where $gc_i = 0.144$ and $V_{syn} = 2.063$ for excitatory edges, and $gc_i = 0.148$ and $V_{syn} = -1.547$ for inhibitory edges. The inference results, including the estimations of NNs and the inaccuracy are shown in Sup. Fig. 5:

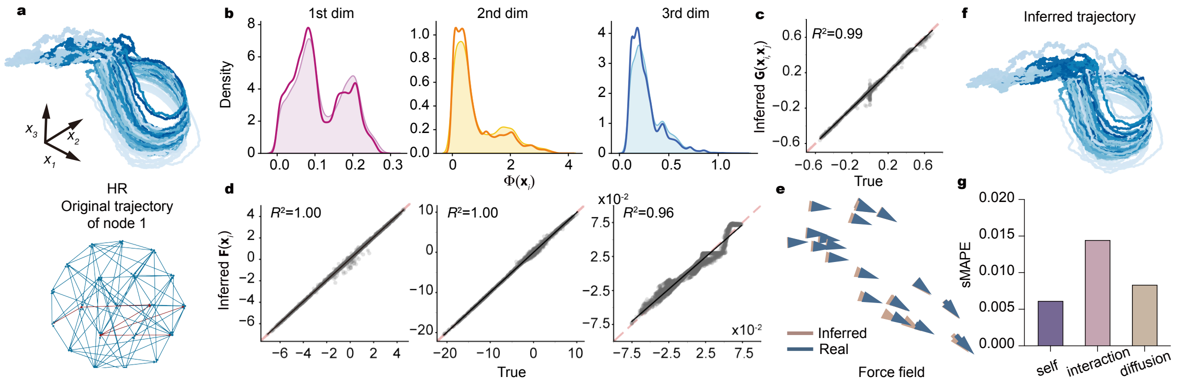

Supplementary Figure 5: **a**. The trajectory simulated by the true stochastic HR dynamics and the weighted network topology. **b**. The inferred distribution density of the diffusion intensity vs. true intensity distribution. **c**. The inferred interaction by $\hat{g}(\cdot)$ vs. the true interaction values. **d**. The inferred 3-dimensional self by $\hat{\boldsymbol{f}}(\cdot)$ vs. the true self values. **e**. Comparison between the generated force field by inferred equation and the true force field at time $t = 10$. **f**. The 3-dimensional trajectory generated by the inferred stochastic HR equation. **g**. Inference inaccuracy sMAPE of the inferred stochastic HR dynamics.

B.4 Second-order stochastic dynamics of Vicsek flocking model

In the final simulation case, we verify the ability to infer the 2nd-order stochastic dynamics, *i.e.* Vicsek flocking dynamics. The Vicsek dynamics is described as above[S7]:

$$\dot{\mathbf{v}}_i = \gamma(v_0^2 - |\mathbf{v}_i|^2)\mathbf{v}_i + \sum_{j \neq i}(\mathcal{C}(r_{ij})\mathbf{r}_{ij} + \mathcal{A}(r_{ij})\mathbf{v}_{ij}) + \sigma(\mathbf{v}_i)\mathrm{d}\mathbf{W}_i, \tag{S11}$$

where $\mathrm{d}\mathbf{W}_i \sim \mathcal{N}(0, \mathrm{d}t)$, $\mathrm{d}t = 0.01$. Shown as above, the core of interaction is composed by cohesion strength $\mathcal{C}(r_{ij}) = \epsilon_0(1 - (r/r_0)^3)/((r/r_0)^6 + 1)$ and alignment strength $\mathcal{A}(r_{i,j}) = \epsilon_1 \exp(-r/r_1)$, where $\epsilon_0 = 1.5$ and $\epsilon_1 = 1$. In Eq.S11, $\gamma = 2$, $v_0 = 1.5$, $r_0 = 2$, $r_1 = 3$, $\mathbf{v}_i = \dot{\mathbf{r}}_i$, $\mathbf{r}_{ij} = \mathbf{r}_j - \mathbf{r}_i$, and $\mathbf{v}_{ij} = \mathbf{v}_j - \mathbf{v}_i$. And the diffusion intensity function is $\sigma(\mathbf{v}_i) = \mathbf{v}_i$. Optimizing the loss function:

$$\mathcal{L} = \beta_1\mathcal{L}_{\mathrm{nl}} + \beta_2\mathcal{L}_{\mathrm{r}} + \beta_3\mathcal{L}_{\mathrm{v}} + \beta_4\mathcal{L}_{\mathrm{a}}, \tag{S12}$$

where $\beta_1, \beta_2, \beta_3, \beta_4$ are hyperparameters balancing the different parts of loss, $\mathcal{L}_{\mathrm{nl}}$ is the negative log-likelihood loss, $\mathcal{L}_{\mathrm{r}}$, $\mathcal{L}_{\mathrm{v}}$ and $\mathcal{L}_{\mathrm{a}}$ are the squared error between the predicted and true displacements, velocities and accelerations, respectively. For inferring the simulated Vicsek model, $\beta_1 = 1, \beta_2 = 1e^6, \beta_3 = 1e^6$, and $\beta_4 = 1e^3$. The inferred equation is:

$$\dot{\mathbf{v}}_i = (4.03 - 1.97|\mathbf{v}_i|^2)\mathbf{v}_i + \sum_{j \neq i}(\boldsymbol{c} \cdot (\frac{1 - (r_{ij}/2)^3}{1 + (r_{ij}/2)^6})\mathbf{r}_{ij} + \boldsymbol{a} \cdot (e^{-r_{ij}/3})\mathbf{v}_{ij}) + \mathrm{d}\mathbf{W}_i, \tag{S13}$$

where $\boldsymbol{c} = [1.33, 1.35, 1.34]^{\mathrm{T}}$ and $\boldsymbol{a} = [0.99, 1.00, 0.99]^{\mathrm{T}}$, which correspond to the coefficients of different dimensions for cohesion and alignment intensity. And the inference inaccuracies sMAPE of self, cohesion, alignment and diffusion intensity parts are 0.0953, 0.0563, 0.0034 and 1, respectively.

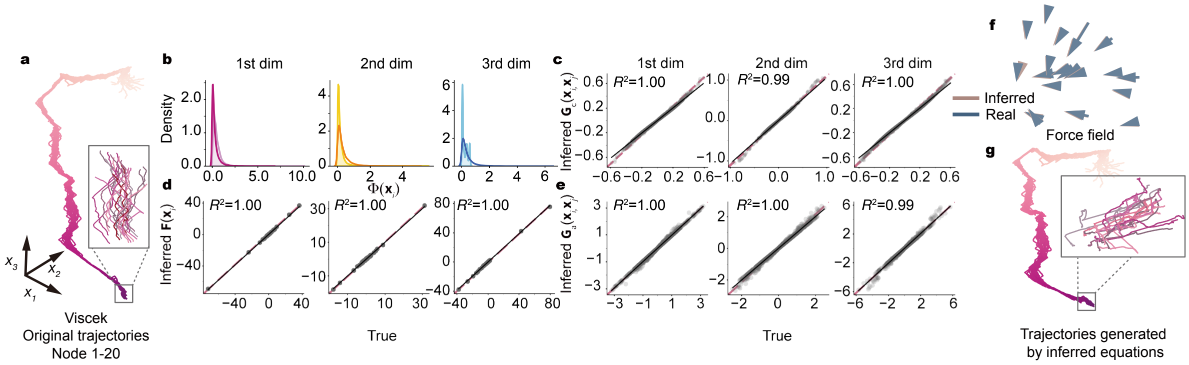

Supplementary Figure 6: **a**. Simulated Viscek stochastic 3-dimensional trajectories of all nodes. **b**. The comparison of inferred and true diffusion intensities distributions. **c,e**. The comparison of inferred and true cohesion and alignment values of 3 dimensions. **d**. The comparison of inferred and true 3 dimensions' self values. **f**. The reproduced force field governed by inferred second-order stochastic Vicsek dynamics comparing with true force field at time $t = 10$. **g**. 3-dimensional trajectories generated by inferred Viscek SDEs.

III Learning from experimental data

A Interpretable dynamics of real bird flocks

As discussed in the main manuscript, the inferred equations of empirical bird flocks [S8] are modified versions of the Vicsek model with alignment strength $\hat{\mathcal{A}} = a_1(\exp(-r_{ij}/3) + a_2) + a_3$, cohesion strength $\hat{\mathcal{C}} = c_1((r_{ij}/2-1)^3/(r_{ij}/2+1)^6 + c_2) + c_3$, and self-propulsion strength $\hat{\mathcal{S}} = s_1(|\mathbf{v}_i|^2 + s_2) + s_3$. We test the strengths in four flock datasets to obtain the scaling parameters, as shown in Sup. Fig. 8 and Sup. Tab. 5 below. The hyperparameters for loss function are $\beta_1 = 1, \beta_2 = 1e^6, \beta_3 = 1e^6$, and $\beta_4 = 1e^6$. The surfaces of functions of the empirical flock dynamics are shown in main text, and the surfaces of original Vicsek model are shown in the following:

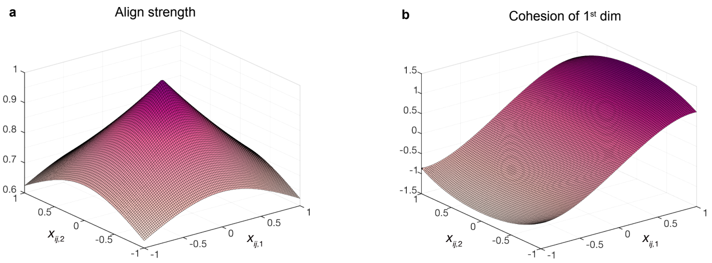

Supplementary Figure 7: **a**. The surface of align strength. **b**. The surface of cohesion strength of the first dimension.

B Interpretable dynamics of tau pathology propagation

As mentioned in the main text, the misfolded paired helical filament (PHF) tau was obtained from Alzheimer's disease (AD) patients' brains and the pathological tau was extracted and injected into mouse brains [S9]. The biological operations are described in the main text. Before feeding the pathology diffusion data to the LaGNA, we organized and expanded it. First, we matched the neuroanatomical connections (annotated according to Allen Brain Atlas (ABA)

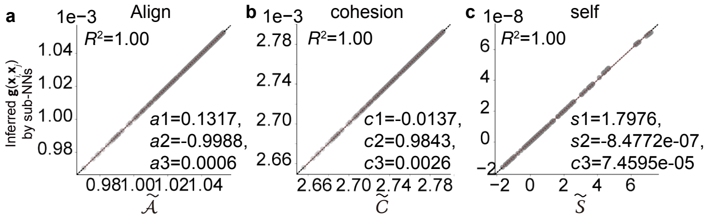

Supplementary Figure 8: **a**. The regression accuracy of estimated alignment intensity comparing with the scaled $\tilde{\mathcal{A}}$, which the parameters are shown in the figure. **b, c**. The regression accuracy of estimated cohesion and self-propulsion intensity comparing with the scaled $\tilde{\mathcal{C}}$ and $\tilde{\mathcal{S}}$, which the parameters are also shown in the figure.

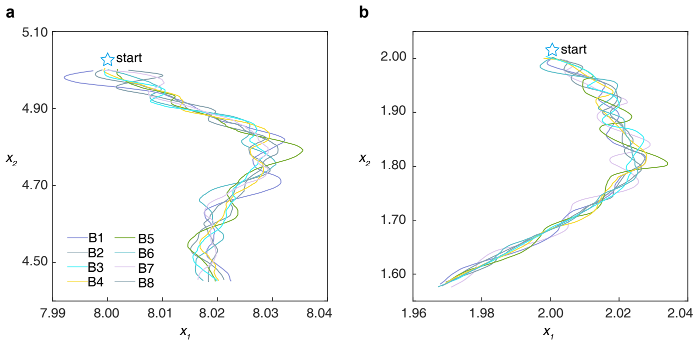

Supplementary Figure 9: Example trajectories (**a** and **b**) generated by the inferred SDE of flocking dynamics with different initial states and coefficients.

Common Coordinate Framework) with the pathology diffusion data. Some areas were missing from the pathology dataset. For example, if area iAAA was missing, we ignored this area in the neuroanatomical connections matrix. If some areas were missing, but the homologous region's data was existing (e.g., area iORB), we simply set the sub-regions iORBl, iORBm, and iORBvl's tau pathology level to be the same as iORB's tau pathology. This way, we matched the connection matrix and corresponding pathology dataset. Second, since the original dataset was constructed from 24 mice's pathology, and we wanted to discover the average diffusion dynamics, the data amount was relatively small. We extended the data by randomly selecting a small offset near the mean value of each observation moment 5000 times. The offset was related to the standard deviation of the real data at that moment.

The processed data are fed into the LaGNA which is trained with a learning rate of $lr = 1e-6$. After thorough training, the LaGNA is able to capture the effective underlying dynamics implicitly, and the predictions of these effective dynamics are shown in Sup. Fig. 10. And by fine-tuning, the coefficients in the inferred equation are $c_t = [-1.56, -6.16, -18.32, -27.90]$ for 1, 3, 6, and 9 months post-injection (MPI).

It should be noted that in our model, we consider both neuroanatomical connections and Euclidean distance. However, we assume that only extremely close areas could rupture and permeate pathology. This assumption is validated by the following test: as shown in Sup. Fig. 11, the prediction accuracy is higher when considering interactions only happening in extremely close Euclidean distance (Sparse, 10% links are kept) compared to considering the complete Euclidean distance (Complete). We also simulated the tau pathology diffusion according to the heterogeneous model (given the initial injection states), and the predictions of all areas in the brain are shown in Sup. Fig. 14-16.

Parameters	1st	2nd	3rd	4th
a1	0.132	0.603	-0.203	0.004
a2	-0.999	-0.995	1	-0.997
a3	0.001	-0.003	0	0.001
c1	-0.014	-0.007	0.186	-0.107
c2	0.984	0.940	1	0.963
c3	0.003	0	0.004	0.001
s1	1.798	1.959	6.167	0.291
s2	0	0	0	0
s3	0	0	0	0

Supplementary Table 5: The scaling parameters for each flock dataset.

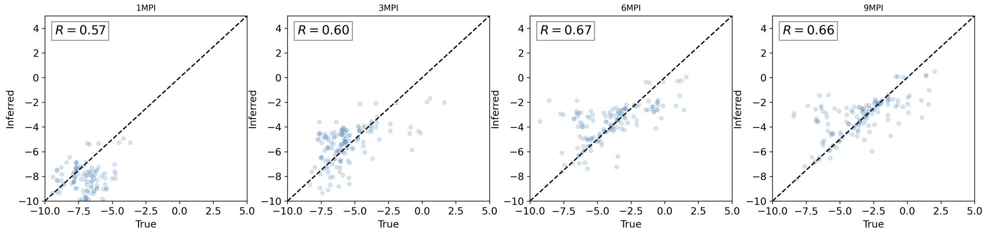

Supplementary Figure 10: Predictions vs. true diffusion of effective dynamics. R is Pearson correlation coefficient.

C Details of the penetration procedure

Once our LaGNA model is well-trained, we proceed to penetrate the self, interaction, and diffusion modules respectively. The penetration procedure involves two steps: (1) a global regression to determine the minimal number k of required terms, and (2) a fine-tuning analysis to identify the specific terms. During these steps, we do not set a hard threshold but make decisions based on regression curves. Below, we use the AD propagation data as an example to detail the penetration procedure.

In step 1, we identify the top 5 terms with largest regression coefficients and sequentially add them to the equation, then calculate the regression score (R^2) and Akaike Information Criterion (AIC) of the equation with one to five terms respectively. For example, as shown in Figs. 12-left and 13-left, adding only one term (except the 'constant') with the largest coefficient already leads to a regression score larger than 0.9. Further addition of the remaining terms does not significantly increase the regression score or significantly decrease of the AIC. Hence, the number k of required term is determined to be one (except the 'constant').

In step 2, we assess each combination of k terms in the top 5 preliminary terms:

• If a single combination has a significantly higher regression score than other combinations, that combination of terms is finally selected for the equation. For example, in Fig. 12-right (where $k = 1$), among the four terms (*i.e.*, Exp, Tanh, Hill, Sigmoid), we find that the sigmoid term achieves the highest regression score and is therefore selected as the expression for the retrograde interaction dynamics in the AD propagation data.

• If multiple combinations achieve similar high regression scores, we select the term with the lowest complexity. For example, in Fig. 13-right (where $k = 1$), the four terms achieve similar regression scores individually. Hence, we proceed to a simple step 3 to analyze the complexity.

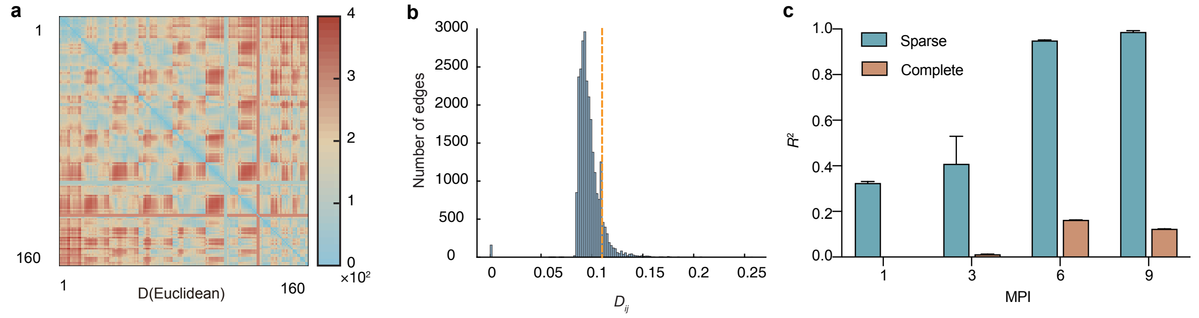

Supplementary Figure 11: Comparison of sparse and complete Euclidean distance models' predictions accuracy. **a**. Euclidean proximal connections. **b**. Frequency distribution of Euclidean Distances among Various Brain Regions. **c**. The prediction accuracy produced by sparse Euclidean proximal network and the fully-connected Euclidean proximal network, with error bars indicating the standard deviation of five individual predictions.

As shown in Fig. 13 (Step 3), each term can be represented by a binary tree, and the complexity of a mathematical expression is defined as $Complexity = D + N_{\text{leaf}}$, where D is the depth of the tree and N_{leaf} is the number of leaves in the tree. Since the Exp term has the lowest complexity, it is selected to capture the spatial dynamics in the AD propagation data.

After determining the necessary terms, we obtain the coefficient for each term by regressing the observed time series data. Note that the constant term is assessed separately. We found that adding a constant to the interaction dynamics of the inferred equation does not significantly increase the final regression score for AD propagation. Consequently, the inferred equation (Eq. 5 in the main paper) does not include a constant term in the retrograde, anterograde, or spatial propagation parts.

In summary, during the penetration procedure, we first determine the necessary terms and then obtain their coefficients. Since our objective is to infer the most concise SDE from the data, we prioritize having a minimal number of terms and those with the lowest complexity.

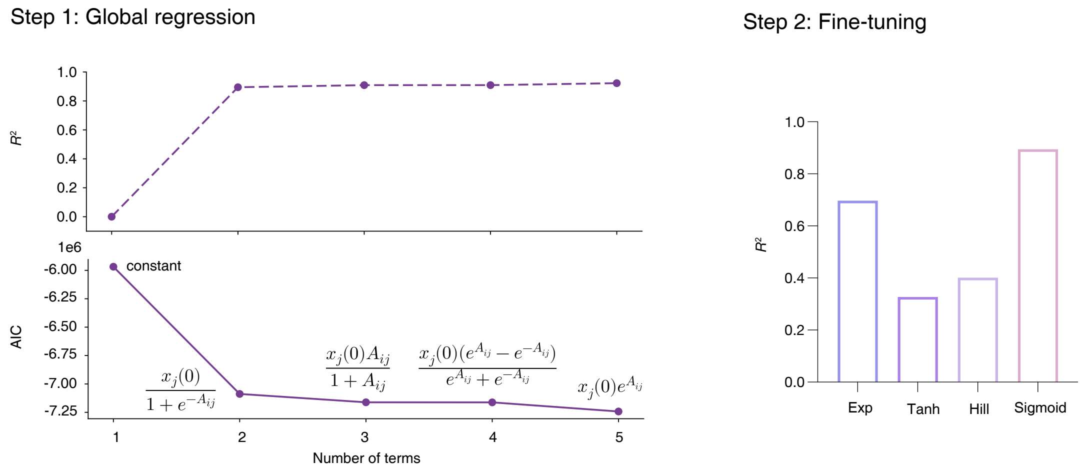

Supplementary Figure 12: Identifying the terms to represent the retrograde interaction dynamics in AD propagation.

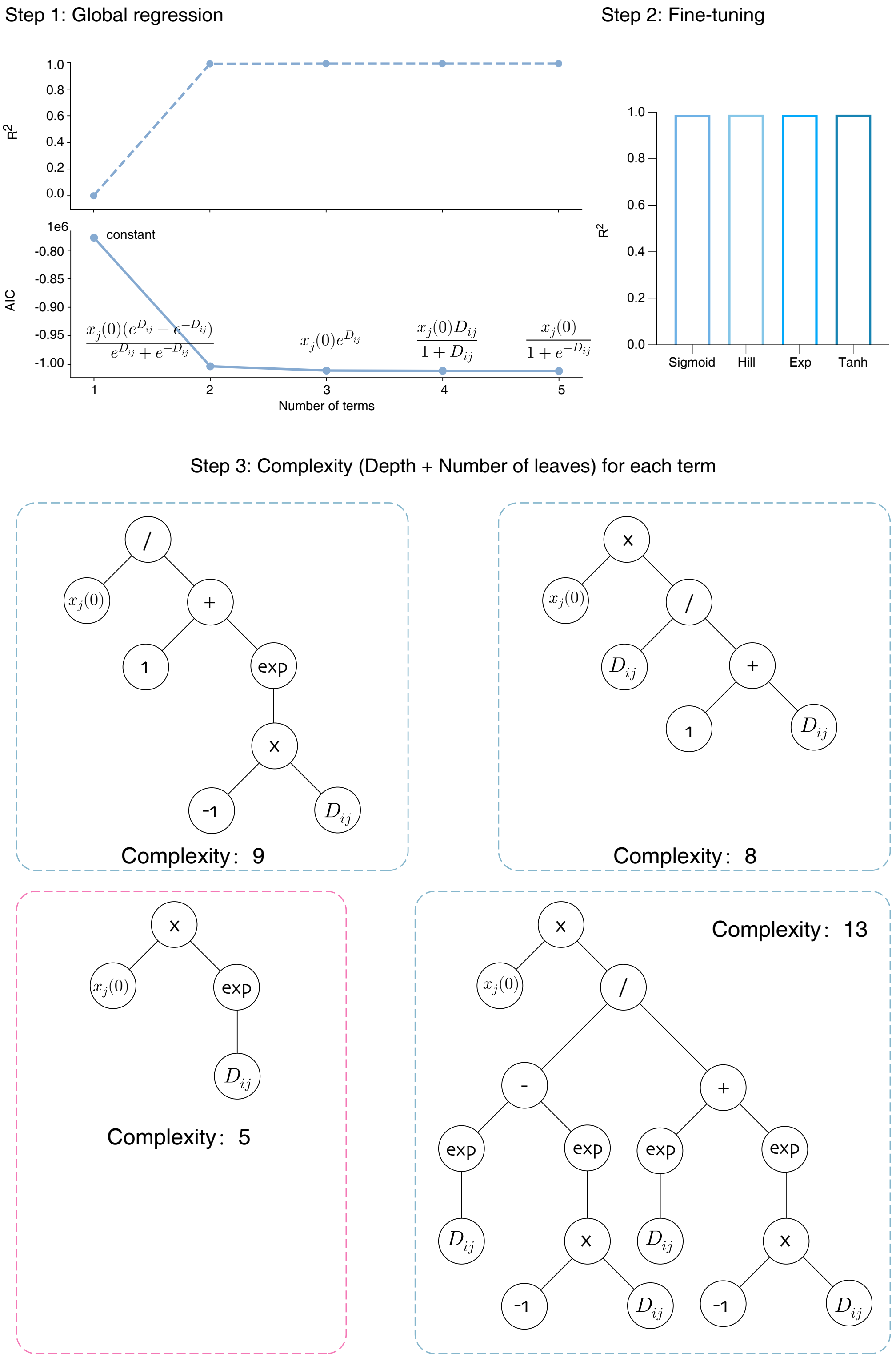

Supplementary Figure 13: Identifying the terms to represent the spatial interaction dynamics in AD propagation.

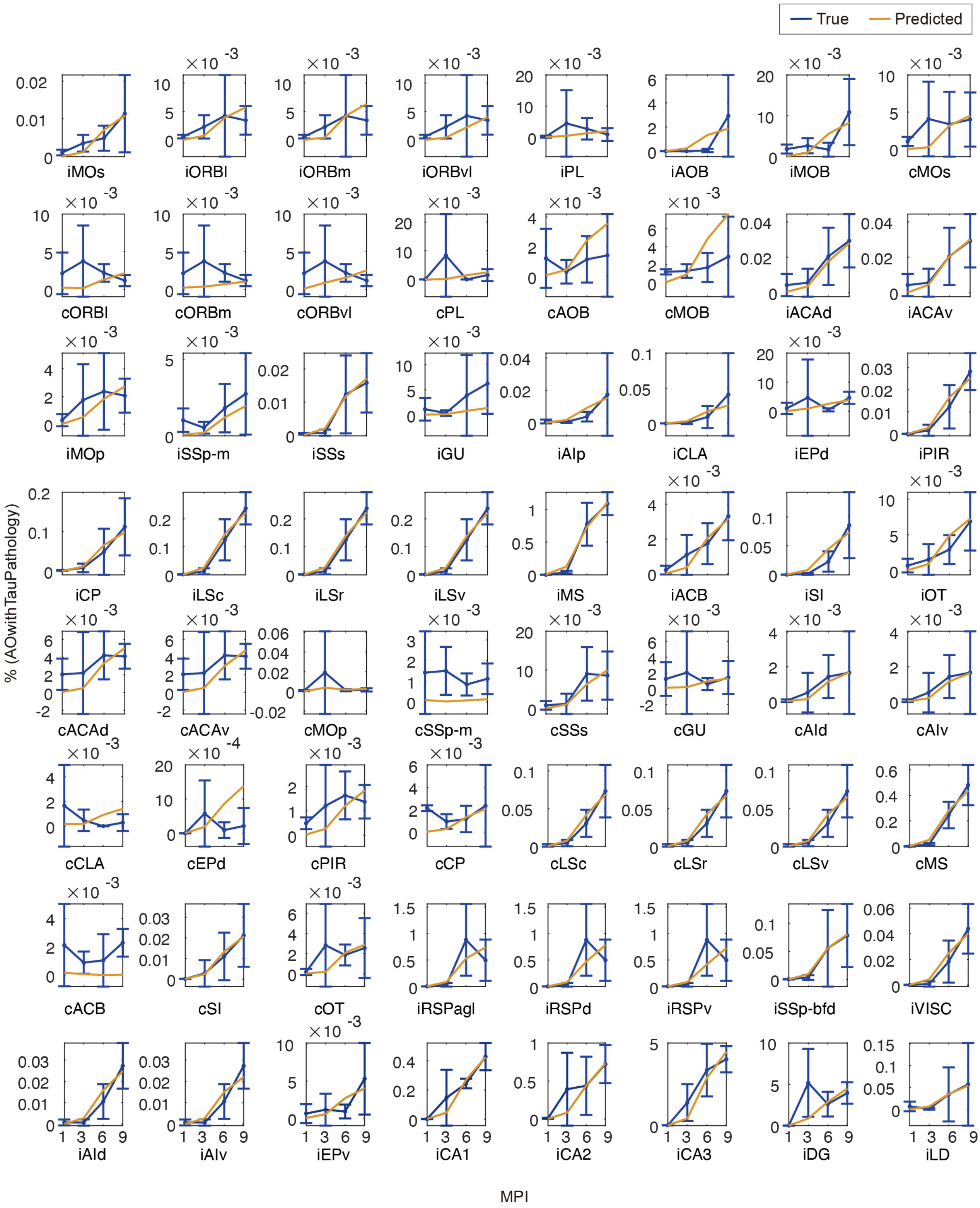

Supplementary Figure 14

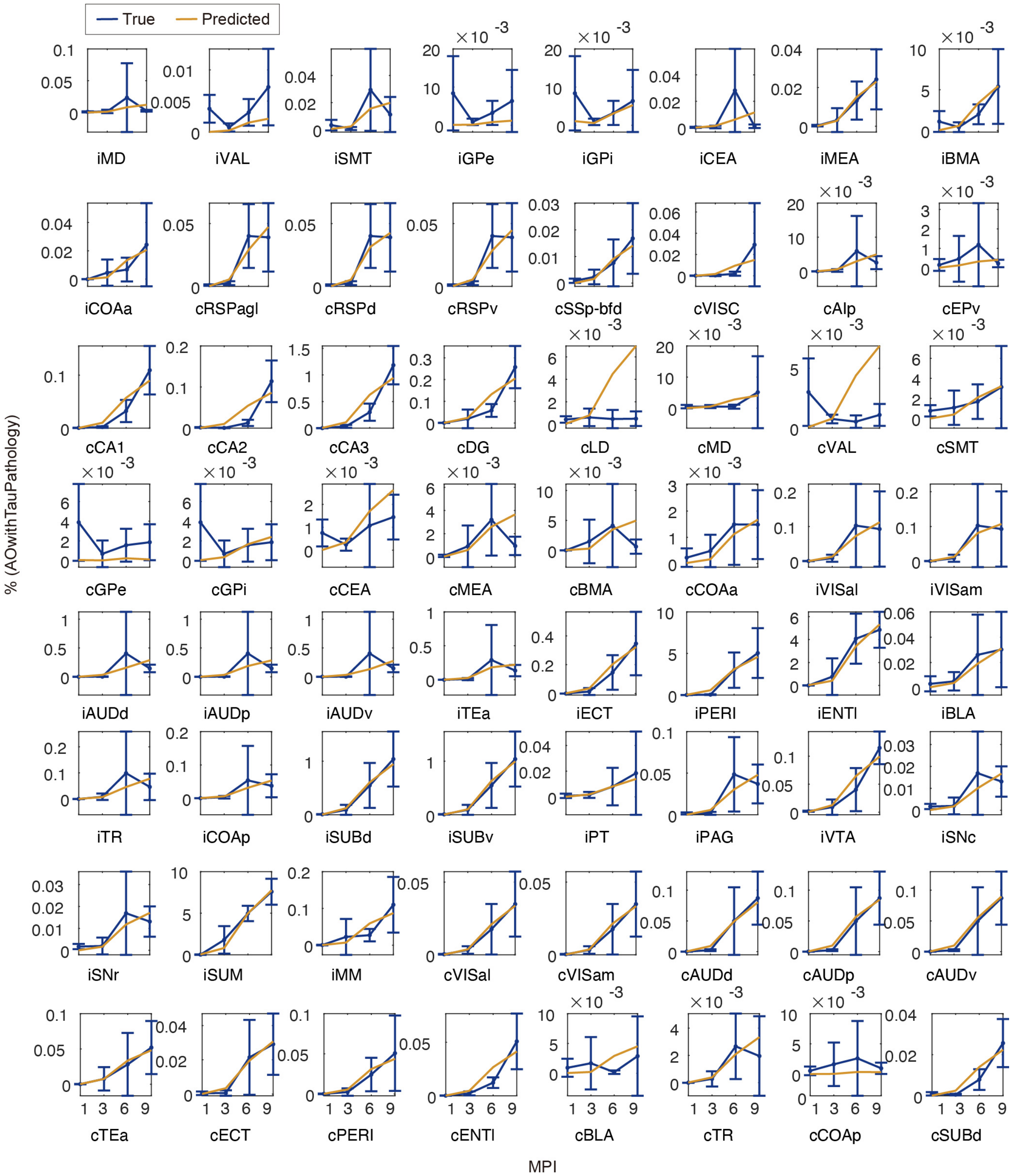

Supplementary Figure 15

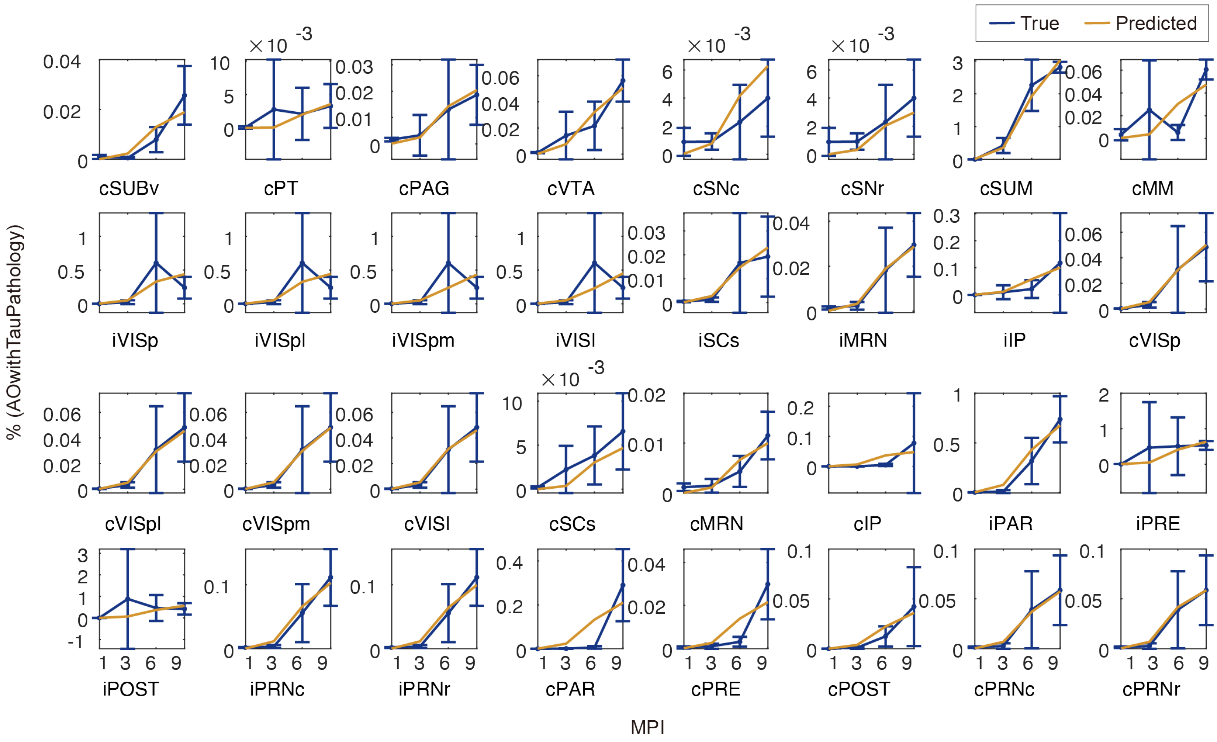

Supplementary Figure 16: Together with Sup. Figs. 14 and 15, showing the comparison between true and predicted area occupied (AO) ratio with tau pathology in all regions.

IV Robustness test of LaGNA

To validate the performance of our framework, in this section, we designed two robustness tests. During the training process, as shown in Fig. 17 (a,b), the regression trajectories and probability density function (PDF) gradually approached the ground truth and tended to become stable with iterative training. Based on this observation, we trained the NNs with 30-50 epochs.

It is worth noting that the first perturbation set for the robustness test was customized for the core mechanism of message-passing type GNN. A message-passing type GNN propagates node features by exchanging information according to the inputted adjacency matrix. Therefore, introducing an incomplete adjacency matrix leads to the loss of part of message propagation. We gradually adjusted the percentage of missing links by randomly choosing and deleting existing edges, and the inference inaccuracy was quantified by sMAPE [S10]. The range of sMAPE is $[0, 1]$, and the more accurate the inferred equation, the lower the value of sMAPE. For stochastic Rossler oscillators, the tolerance of missing links is 3%, while for signed HR and Lorenz stochastic systems, the tolerance of missing links is both around 15%, which is unexpectedly high. The variable settings for the simulation of the above three stochastic systems are the same as the examples in the main text.

The other challenging situation is signed networks showing heterogeneity, which is a typical characteristic of biological neuronal networks. The previous two-phase network inference approach is able to discover the exact elementary functions of homogeneous network dynamics, however, it is difficult to identify the two types of interaction functions because of the combination effect, *i.e.* the interaction part can be summed by excitatory and inhibitory effects, the blur may be represented by alternative functions instead of two types of functions. The challenge will also happen in stochastic dynamics. Thanks to the structural flexibility of our framework, we set two NNs for two types of interactions. We demonstrate the ability to infer excitatory and inhibitory elementary functions by adjusting the proportion of inhibitory edges, and the results show that the closer the ratio of two types of edges is to $1:1$, the more accurate the inferred SDEs will be.

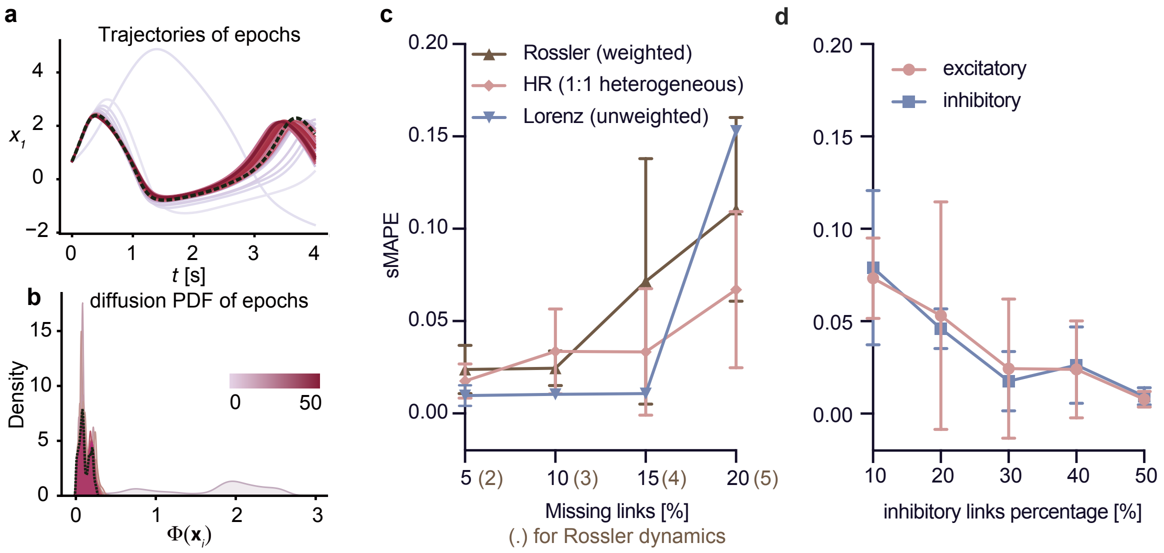

Supplementary Figure 17: **a**. The trajectories of the first node's 1st dimension generated by drift NNs, *i.e.* only numerically integrate using $\tilde{\mu}$, get closer to the true deterministic trajectory (black dashed line) with more iterations. **b**. As the iterations the estimated diffusion intensities distribution get close to the real distribution. **c**. Inference inaccuracy sMAPE increases when the network topology misses links, for Rössler dynamics, the x-coordinate ranges in $[2, 5]$, and for HR and Lorenz range in $[5, 20]$. **d**. Inference inaccuracy sMAPE decreases under the different proportions of excitatory (or inhibitory) type of edges situations.

V Additional comparisons

A Description of baseline methods

Here we briefly describe the related methods and how they were extended to infer the hidden SDEs of complex networked systems for comparison purpose.

Modified-SINDy [S11]: a sparse identification method designed specifically for time series data characterized by significant observational noise. The Modified-SINDy method possesses the capability to discern the underlying equations from noisy data and can achieve high accuracy in estimating the distribution of observational noise. Although primarily developed to ascertain the dynamics of single-node systems, this method can be extended to infer complex network dynamics by condensing topological information within coupling terms [S1]. In the comparative testing, we included coupling functions, such as $\sum_{j=1}^{n} x_j$ and $\sum_{j=1}^{n}(x_j - x_i)$ in the library of Modified-SINDy. By applying Modified-SINDy to learn the SDEs from observed data of a networked stochastic Lorenz system, we obtained the sparse identification results and calculated the inference error using sMAPE.

SVISE [S12]: a method for state estimation of noisy and latent observations, without prior knowledge of the governing equation. State estimation can be performed based on a small amount of data, and equation identification can be conducted for low-dimensional systems. For high-dimensional systems, SVISE has a module called neuralSDE to meet the demand. Similar to the extension of Modified-SINDy, SVISE can incorporate topological information into the elementary function library by adding coupling functions, making it suitable for networked dynamics inference.

SDE-net [S13]: a method for estimating the drift and diffusion parts of single-node stochastic systems. To perform fair comparison, we extended its drift part to including both self-dynamics and interaction dynamics.

Two-Phase inference [S1]: our previous method for inferring the hidden ODEs of complex networked systems from noisy and incomplete data. No improvements have been made in this method.

Stochastic force inference (SFI) [S14]: a method for inferring stochastic force, applicable to both monomer stochastic systems and homogeneous multi-particle systems. The core idea in SFI is kernel regression. Similar to SDE-net, SFI estimates the drift and diffusion parts from the observed data by using the combinations of kernel basis.

The comparative tests are conducted on the benchmark system, i.e., the networked stochastic Lorenz system whose ground-truth SDEs are known as Eq. S4. The comparison results are shown in Fig. 2m in the main paper. It is worth noting that SVISE can still accurately estimate the system states from the data but cannot obtain the accurate governing equation; SDE-net and SFI appear to be able to identify the drift and diffusion parts but unable to separate the self- and interaction dynamics. As SFI uses a series of kernel functions, its inference result can make accurate predictions but does not offer a concise and interpretable mathematical expression.

S-OnsagerNet [S15]: a deep learning method specifically designed for constructing *macroscopic* stochastic dynamics from nodal trajectories [S15]. Because its objective and the dimension of its output is distinct from ours and other five methods, S-OnsagerNet cannot be modified for comparison.

B Learning from noisy data with a denoising preprocess

Empirical and experimental observations often exhibit a combination of intrinsic stochasticity and extrinsic noise, with the latter arising from measurement errors. Distinguishing between intrinsic and extrinsic noises can pose challenges [S16]–[S18]. In cases where no prior knowledge is available regarding the dominant source of noise in a given set of real data, we treat all noises as intrinsic, an assumption we made in the present work.

In this subsection, we examine the challenge posed by extrinsic noise to our LaGNA method and explore the impact of adding a denoising preprocess. Illustrated in Sup. Fig. 18, we take the networked Lorenz system with an intrinsic stochasticity intensity of $1/\sqrt{\gamma} = 1$ as an example. We introduce observational noise $\sigma p \nu(t)$ into the data, where σ represents the standard deviation of the original stochastic time series, $\nu(t)$ is a random number drawn from a Gaussian distribution with a mean of zero and a variance of one for time step t, and $p \in [0, 1]$ denotes the relative strength of observational noise [S11]. The outcomes reveal that when treating all noises as intrinsic, our method LaGNA demonstrates accurate inference of the stochastic dynamics of the networked system when the relative strength of observational noise is below 10%. However, when the observational noises are more pronounced, the preprocessing step of distinguishing between intrinsic and extrinsic noises (in this case, we used the Kalman-Takens filter [S18]) proves beneficial in enhancing the capability of our method. This enhancement becomes particularly notable when inferring the stochastic dynamics of single-node systems (as depicted in Sup. Fig. 19 below).

	Single-node ODE learning		Single-node SDE learning		Multi-nodes ODE learning		Multi-nodes SDE learning	
	Capability	Interpretability	Capability	Interpretability	Capability	Interpretability	Capability	Interpretability
Modified-SINDy [S11]	✓	✓	×	×	✓	✓	×	×
SVISE-sparse [S12]	✓	✓	✓	✓	×	×	×	×
SVISE-SDE-NN [S12]	-	-	✓	×	✓	×	✓	×
SDE-net [S13]	-	-	✓	×	-	-	×	×
S-OnsagerNet [S15]	-	-	-	-	-	-	✓	-
Two-Phase [S1]	✓	✓	×	×	✓	✓	×	×
SFI [S14]	-	-	✓	×	-	-	✓	×
**LaGNA**	✓	✓	✓	✓	✓	✓	✓	✓

Supplementary Table 6: Objectives and applicable scenarios of the methods. Symbol ✓indicates that the objective of the method itself is consistent with the problem, or it can be used for accurate inference with simple modifications; Symbol × indicates that the method can be applied to this problem after major modifications, but the accuracy is low (i.e., sMAPE is higher than 0.5); Symbol - indicates that the objective is completely different from the problem and the method cannot be modified for comparison.

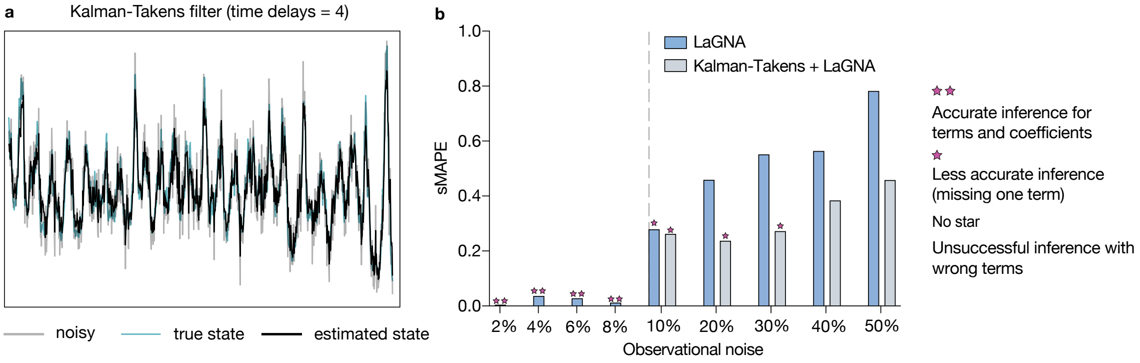

Supplementary Figure 18: **a**. Denoising by Kalman-Takens filter. **b**. Comparison of inference inaccuracy (sMAPE) under different observational noise intensities.

C Inferring single-node stochastic dynamics from noisy data

For comparison purpose, we also test the effectiveness of different inference methods on the single-node stochastic system with observational noise. Similar to the last subsection, we introduce observation noise $\sigma p\nu(t)$ to the single-node Lorenz system with an intrinsic stochasticity intensity of $1/\sqrt{\gamma} = 1$. As illustrated in Sup. Fig. 19, our method LaGNA is capable of inferring the SDE of system dynamics when the observation noise is at the level of 10%, and it outperforms the five existing methods. Moreover, for both levels (10% and 50%) of observational noises, the preprocessing step of the Kalman-Takens filter consistently enhances the capability of our method LaGNA.

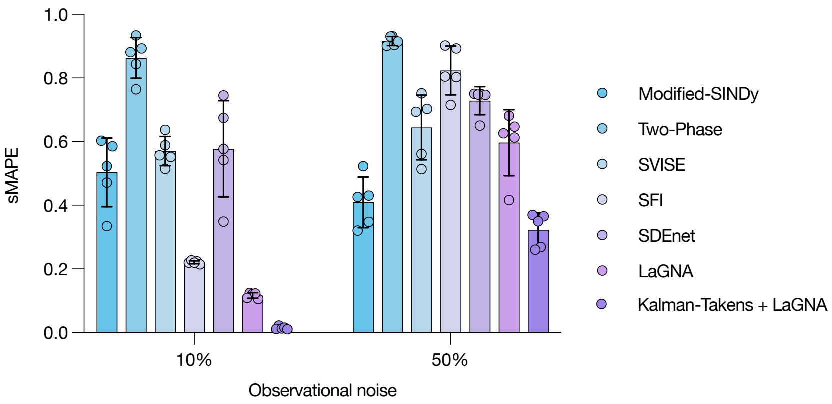

Supplementary Figure 19: Inference inaccuracy (sMAPE) of different methods in inferring the SDE of single-node Lorenz system when the relative strength of observational noise is 10% or 50%. The bar chart depicts the sMAPE of five independent runs, along with the standard deviation indicated

D Method comparisons for AD propagation data

Finally, we conduct an additional comparative study on the AD propagation dataset. Specifically, we implement three existing methods for inferring stochastic systems: SFI, SDE-net, and SVISE. To ensure a fair comparison, we extended these methods to apply to complex networked systems (refer to Sec. V-A). Using the inferred equations from these methods, we generate propagation trajectories based on the initial state of injected regions. The correlation between the predicted and the true pathologies is shown in Sup. Fig. 20. The results demonstrate that

the equations inferred by these three existing methods have significantly less predictive power compared to our method (Sup. Fig. 10).

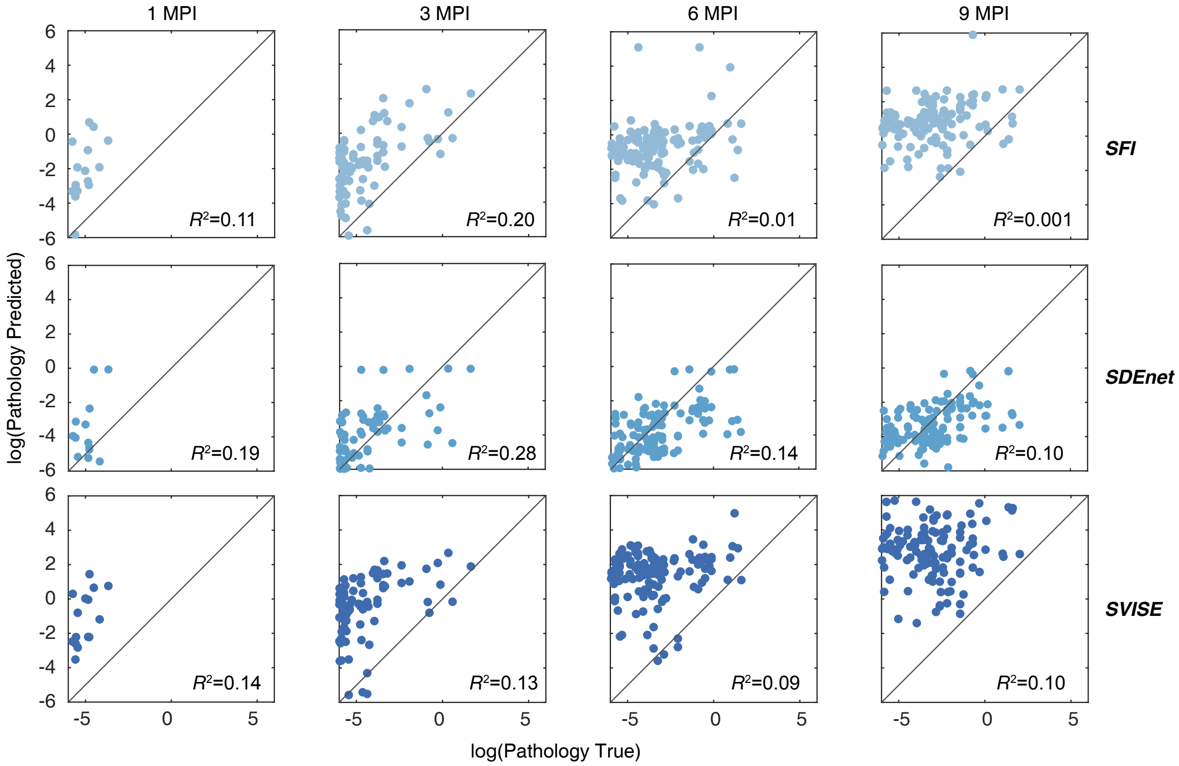

Supplementary Figure 20: The predictions of tau pathology from the equations inferred by SFI, SDE-net, and SVISE, compared to the true tau pathology at 1, 3, 6, and 9 MPI respectively. The square of Pearson correlation R^2 for multiple linear regression tests are noted.

Supplementary References

[S1] Gao, T.-T. & Yan, G. Autonomous inference of complex network dynamics from incomplete and noisy data. *Nat. Comput. Sci.* **2**, 160–168 (2022).

[S2] Goh, K.-I., Kahng, B. & Kim, D. Universal behavior of load distribution in scale-free networks. *Phys. Rev. Lett.* **87**, 278701 (2001).

[S3] Platen, E. & Bruti-Liberati, N. *Numerical solution of stochastic differential equations with jumps in finance*, vol. 64 (Springer Science & Business Media, 2010).

[S4] Dridi, N., Drumetz, L. & Fablet, R. Learning stochastic dynamical systems with neural networks mimicking the euler-maruyama scheme. *EUSIPCO 2021* 1990–1994 (2021).

[S5] Arenas, A., Díaz-Guilera, A., Kurths, J., Moreno, Y. & Zhou, C. Synchronization in complex networks. *Phys. Rep.* **469**, 93–153 (2008).

[S6] Copelli, M. *et al.* Oscillations and collective excitability in a model of stochastic neurons under excitatory and inhibitory coupling. *Phys. Rev. E* **100**, 062416 (2019).

[S7] Brückner, D. B., Ronceray, P. & Broedersz, C. P. Inferring the dynamics of underdamped stochastic systems. *Phys. Rev. Lett.* **125**, 058103 (2020).

[S8] Nagy, M., Ákos, Z., Biro, D. & Vicsek, T. Hierarchical group dynamics in pigeon flocks. *Nature* **464**, 890–893 (2010).

[S9] Cornblath, E. J. *et al.* Computational modeling of tau pathology spread reveals patterns of regional vulnerability and the impact of a genetic risk factor. *Sci. Adv.* **7**, eabg6677 (2021).

[S10] Flores, B. E. A pragmatic view of accuracy measurement in forecasting. *Omega* **14**, 93–98 (1986).

[S11] Kaheman, K., Brunton, S. L. & Kutz, J. N. Automatic differentiation to simultaneously identify nonlinear dynamics and extract noise probability distributions from data. *Mach. learn.: sci. technol.* **3**, 015031 (2022).

[S12] Course, K. & Nair, P. B. State estimation of a physical system with unknown governing equations. *Nature* **622**, 261–267 (2023).

[S13] Dietrich, F. *et al.* Learning effective stochastic differential equations from microscopic simulations: Linking stochastic numerics to deep learning. *Chaos* **33** (2023).

[S14] Frishman, A. & Ronceray, P. Learning force fields from stochastic trajectories. *Phys. Rev. X* **10**, 021009 (2020).

[S15] Chen, X. *et al.* Constructing custom thermodynamics using deep learning. *Nat. Comput. Sci.* **4**, 66–85 (2024).

[S16] Swain, P. S., Elowitz, M. B. & Siggia, E. D. Intrinsic and extrinsic contributions to stochasticity in gene expression. *Proc. Natl Acad. Sci.* **99**, 12795–12800 (2002).

[S17] Lind, P. G. *et al.* Extracting strong measurement noise from stochastic time series: Applications to empirical data. *Phys. Rev. E* **81**, 041125 (2010).

[S18] Hamilton, F., Berry, T. & Sauer, T. Kalman-takens filtering in the presence of dynamical noise. *Eur. Phys. J. Spec. Top.* **226**, 3239–3250 (2017).
